# Changes in the predominant human *Lactobacillus* flora during in vitro fertilisation

**DOI:** 10.1186/1476-0711-7-14

**Published:** 2008-06-30

**Authors:** Tell Jakobsson, Urban Forsum

**Affiliations:** 1 Department of Clinical and Experimental Medicine, Clinical Microbiology, Linköping University, SE-581 85, Linköping, Sweden

## Abstract

**Background:**

Signature matching of nucleotide sequences in the V1 and V3 regions 16S rRNA genes using pyrosequencing technology is a powerful tool for typing vaginal Lactobacilli to the species level and has been used for investigating the vaginal microbial niche.

**Methods:**

This study has characterized the normal cultivable vaginal *Lactobacillus *flora at varying estradiol levels in plasma; the study comprised 17 patients undergoing ovarian stimulation for In Vitro Fertilization (IVF) treatment. The vaginal status of each participant was initially assessed as normal according to Amsel and Nugent criteria.

**Results:**

*L. crispatus, L. gasseri and/or L. jensenii *were present in 10 of the patients throughout the study period, and little variation among these three species was encountered in individual patients. The flora of three women was dominated by *L. delbrüeckii, L. rhamnosus or L. vaginalis*. One woman exhibited a dominance of *L. iners*. The flora of the remaining three women were initially dominated by *L. rhamnosus *or *L. reuteri*, but as their estrogen levels rose, their flora composition altered, to become dominated by one of the three species most common in a normal, healthy vagina.

**Conclusion:**

Signature matching of nucleotide sequences in the V1 and V3 regions of 16S rRNA genes is a discriminative tool for the study of vaginal *Lactobacilli *and can be used to track the *Lactobacillus *flora under a variety of physiological conditions.

## Background

For more than a century, science has known that the Döderlein bacilli dominate the observable microflora of the normal healthy vagina of women of reproductive age; microscopic studies show Gram-positive rods to be predominant. An altered cultivable flora arises when the number of lactobacilli in the vaginal fluid drastically decreases, and opportunistic bacteria, normally present in minute proportions, overgrow the vaginal epithelium. This leads to a rising pH (>4,7) which is conducive to the growth of anaerobes [1]. Maintenance of the normal vaginal flora is supposedly essential because it not only occupies the epithelial surface, but also constitutes a milieu hostile to attacks of pathogens [2]. A prevailing assumption is that the dominating normal vaginal flora comprises the genus *Lactobacillus *more specifically now known as the *L. acidophilus *complex. This assumption is based on earlier literature describing studies using phenotypic methods for typing bacteria and a line of reasoning about the bacterial ecology of normal niches in humans [1].

To acquire further insight into microbial variation of normally occurring bacterial population in defined biological niches, it is important to look closely at the physiology of the particular niche [3]. Conditions making up the niche must be defined. The clinical criteria of Amsel [4] and the scoring system of Nugent [5] provide means for categorizing vaginal flora as normal or otherwise, though mainly in relation to the clinical entity bacterial vaginosis (BV) [6]. Certain physiological conditions are generally assumed necessary if lactobacilli are to thrive in the healthy vagina of a woman of childbearing age. One such factor is the glycogen contents of the vaginal epithelium that co-variates with estrogen levels. During stimulation for *in vitro *fertilization (IVF), estrogen levels can range from extremely low at the start of treatment to high during pregnancy. This provides an opportunity, to study vaginal flora variation in circumstances that mirror the normal hormonal variation occurring a woman's lifetime. We have previously published studies on healthy women whose vaginal flora status is deemed normal according to Amsel and Nugent [6,7]. Using traditional phenotypic methods, as well as genotypic methods, we confirmed and extended earlier studies implicating *L. crispatus, L. gasseri, L. jensenii *[1,8], and adding *L. iners *[9] to the list of vaginal *Lactobacilli *which normally dominate. The importance of a true random sample to study the dominant vaginal flora in contrast to bacterial strains from culture collections was also made clear [7]. In a second study [6], we used PCR with pyrosequencing followed by signature matching with published type strain sequences as the chosen method to identify those Gram-positive rods constituting the dominant vaginal flora. Both studies were performed on a defined population of fertile, healthy women scheduled for a regular PAP smear. In the first study, three colonies were selected from each sample, in the second, up to ten colonies were studied from each cultured sample. Both studies showed dominance by *L. crispatus, L. jensenii *and/or *L. gasseri*, and in some cases, the newly described, *L. iners*. These findings agree with reports from other parts of the world, even those studies having poorly defined inclusion criteria and those including strains from culture collections [1]. Our research also shows that three randomly collected colonies will represent the dominating flora.

Using a similar approach and definitions according to Amsel and Nugent criteria, this study aims at further characterizing the normal cultivable vaginal flora present at differing estradiol levels in plasma during ovarian stimulation for IVF treatment.

## Material & Methods

The population was recruited from patients with uneventful medical and STD history attending the Reproductive Medicine Centre, Dept of Obstetrics and Gynecology, University Hospital, Linköping, Sweden during January to March 1999 for the final examination prior to IVF stimulation. These women gave their informed consent to participate in the study. Of 34 women who originally agreed to participate, 13 were excluded. In total, the vaginal fluids of 17 patients were cultured on 62 occasions, three to five times per patient. Of the excluded patients, five patients did not fulfill the Amsel and/or Nugent criteria for normal vaginal status, and eight dropped out after one or two visits. Among the patients who returned only once, four exhibited Gram-positive rods growing on horse blood agar, but not on Rogosa agar. Since these strains might nonetheless belong to the dominating normal flora, we included these samples in our analyses.

**Figure 1:**
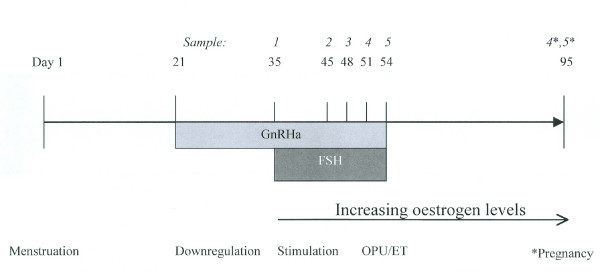
**Protocol of ovulation stimulation in relation to collection of vaginal samples from women undergoing In Vitro Fertilization (IVF) treatment.** GnRH = Gonadotropin Releasing Hormone, FSH = Follicle Stimulating Hormone, OPU = Ovum Pick Up, ET = Embryo Transfer.

The standard IVF protocol (Fig. 1), in accordance with the national Swedish guidelines in effect at the time of the study, started by down regulation with a Gonadotropin Releasing Hormone (GnRH)-analog, nasal administration four times daily, from the 21st day after the start of the menstrual period. Two weeks later, following withdrawal bleeding and a uterine ultrasound that ascertained a minimal endometrium, Follicle Stimulation Hormone (FSH)-stimulation began. After six to eight daily injections, the patient returned to the clinic for an ultrasound check of follicular growth, and thereafter at two to four day intervals until ovum pick-up, and two days after pick-up, for embryo transfer. Six of the patients established a pregnancy and returned for an ultrasound check about six weeks after embryo transfer. At each visit (except at embryo transfer), the level of estradiol in plasma was measured.

In accordance with the protocol, all patients prior to the IVF treatment, ingested prophylactic antibiotics; doxycyclin 200 mg day one, and 100 mg daily for another 8 days, metronidazole 3 times daily for a week. 16 of the patients started IVF treatment 1 to 2 months later. The male partners of the patients were concurrently given the same antibiotic prophylaxis. For six patients, treatment start was further delayed as much as seven months after the antibiotic treatment.

The study was approved prior to its start by the ethics review board for Southeast Sweden.

### Culture

At each visit, vaginal fluid was sampled from the upper third of the vagina by rolling a cotton swab against the lateral wall using a non-lubricated speculum. The swabs were placed in modified Stuart's medium (Copan transport medium, Venturi Transystem, Brescia, Italy) and transported to the laboratory for culture within 3 hours. With another swab, fluid was sampled from the same location and smeared onto a glass slide, which was air-dried, heat-fixed and Gram-stained.

The culture swabs were vortexed briefly in phosphate-buffered saline (PBS), pH 7.2 and diluted in PBS to 1/100, 1/1000 and 1/10 000. One ml of each dilution was plated on horse blood agar and on Rogosa agar (preparation in-house based on Difco media). The plates were incubated for two to four days under microaerophilic conditions (10% CO2 and 5% O2) at 37°C.

### Selection of strains

When growth on Rogosa agar was successful, no colonies were collected from the horse blood agar Petri dishes. If colonies grew only on horse blood agar, they were collected there. Whenever possible, the isolates were picked from the plate with a dilution of 1/10 000 and at a growth of 25 to 100 colonies per dish. To obtain true randomly selected isolates, the dish was reversed and three spots marked with a felt tip pen. When the plate was turned right side up, the spots were clearly visible through the agar. The colony nearest each of the three spots was selected for re-culture under the conditions described above. The colonies were checked for possible impurities with a Gram-stained smear and colony morphology. The bacterial isolates thus obtained were frozen at -70°C in a storage medium based on Nutrient Broth no.2 (Lab M Bury, UK) and 15% glycerol.

### PCR and pyrosequencing

Broad-range PCR followed by pyrosequencing was performed as previously described [6,10].

Primer pairs used for PCR were:

1. bio-pBR 5'.se (position 6–27, E. coli numbering [11]): 5'-biotin-GAAGAGTTTGATCATGGCTCAG-3'; pBR-V1.as (position 120–101): 5'-TTACTCAAAAGTCCGCCACT-3'

2. bio-pJBS-V3.se (position 966–985): 5'-biotin-GCAACGCGAAGAACCTTACC-3';

B-V3.as (position 1065–1046): 5'-AGGTGCTGCATGGCTGTCGT-3'

3. bio-B-V3.as and pJBS-V3.se

Sequencing primers used: pBR-V1.as, pJBS-V3.se and B-V3.as. Primers were obtained from Scandinavian Gene Synthesis, Köping, Sweden.

DNA was prepared with PrepMan™ (Applied Biosystems) lysis reagent. A colony from each isolate was suspended in 50 μl of PrepMan reagent, vortexed, heated in a water bath at 99°C for ten min and centrifuged at 16000 × g for three min. The supernatant was used as DNA template.

The PCR amplification was initially performed in 0.5 ml thin-walled tubes with Ready-to-go beads™ (Amersham Pharmacia, Uppsala, Sweden) on a PTC-100 model thermocycler (MJ Research Inc., Falkenberg, Sweden).

For the major part of the study, PCR was performed in 96-well plates with 5 pmol of each primer, 12.5 μl HotStar Taq (Qiagen, Hilden, Germany), 10.5 μl H_2_O and 2 μl of the prepared DNA template. The PCR was run on a Mastercycler gradient thermocycler (Eppendorf, Hamburg, Germany) with denaturation at 95°C for 15 min, followed by 28–32 cycles of denaturation at 94°C for 40 s, annealing at 55°C for 40 s, extension at 72°C for 1 min; with a final extension step 72°C for 10 min. Agarose gel electrophoresis was performed only on a few samples at the beginning of the study to assure that a PCR product of expected length was obtained.

Pyrosequencing was performed as earlier described [10].

### Bioinformatics

One nucleotide (nt) signature from each sampling was initially screened by comparing with sequences in GenBank using the BLASTn algorithm [12]. Eleven separate categories of signatures were identified, each matching a sequence of a strain deposit in the GenBank library and published in PubMed; our preference was for type strains deposited in ATCC or in certain cases, type strains from other sources. Each category of sequences was compared with previously published reference sequences on vaginal samples (Table 1).

**Table 1:** List of reference strains used to group found signatures of 16s rDNA.

**Species**	**Strain**	**GenBank no**	**Reference**	**Reference 2**
*L. acidophilus*^a^	ATCC4356^T^	AY773947	Kao 2007	Falsen 1999
*L. casei*	ATCC334^T^	NC008530	Makarova 2006	Falsen 1999
***L. crispatus***	KC12a	AF243152	Pavlova 2002	
*L. delbrueckii*	ATCC9649^T^	AY050172	Germond 03	Falsen1999
***L. fermentum***	KC5b(ATCC14931)	AF243166	Pavlova 202	Falsen 1999
*L. gallinarum*	ATCC3319^T^	AJ242968	Ventura 2000	Falsen1999
***L. gasseri***	KC5a(ATCC33323)	AF243165	Pavlova 2002	
***L. iners***	CCUG28746^T^	Y16329	Falsen 1999	Falsen 1999
*L. jensenii*	ATCC25258^T^	AF243176	Pavlova 2002	Falsen 1999
***L. jensenii***	KC36b	AF243159	Pavlova 2002	
***L. jensenii***	KC23	AF243155	Pavlova 2002	
*L. johnsonii*	ATCC33200^T^	AJ002515	Fujisawa 1992	Falsen 1999
***L. mucosae***	BLB1c	AF243145	Pavlova 2002	
***L. paracasei***	KLB58	AF243168	Pavlova 2002	
*L. reuteri*	LU3(DSM20016)	AY735406	Unpubl	Falsen 1999
***L. rhamnosus***	F11(ATCC7469)	AF243146	Pavlova 2002	
***L. vaginalis***	KC19	AF243154	Pavlova 2002	
				
*L. crispatus*^b^	ATCC33820^T^	AF257097	Pavlova 2002	Falsen 1999
*L. kimchii*	AP1077^T^	AF 183558	Yoon 2000	
*L. paraalimentarius*	TB1^T^	AB018528	Cai 1999	
*L. paracasei*	DSM5622^T^	D79212	Mori 1997	Falsen 1999
*L. vaginalis*	ATCC49540^T^	AF243177	Pavlova 2002	Falsen 1999
*L. acidophilus*	NCFM	NC006814	Altermann 2005	
*L. brevis*	ATCC367^T^	NC008497	Makarova 2006	
*L. delb. sp. bulgaricus*	ATCC11842^T^	NC008054	Van de Guchte 2006	
*L. gasseri*	ATCC33323^T^	NC008530	Makarova 2006	
*L. johnsonii*	NCC533	NC005362	Pridemore 2004	
*L. plantarum*	WCFS1	NC004567	Kleerebezem 2004	
*L. reuteri*	F275	NC009513	unpublished	
*L. sak. sakei*	23K	NC007576	Chaillou 2005	
*L. sal. salivarius*	UCC118	NC007929	Claesson 2006	

a/Selected reference strains (**vaginal samples**, in bold, chosen if existing)

b/Additional culture collection strains (of other than vaginal or no information as to origin) with sequences similar to the ones above and whole genome sequenced strains of lactobacilli.

Other published sequences from *Lactobacillus *outside the vaginal niche used for comparison of sequences are listed separately, among them, all whole genome sequences of *Lactobacillus *species published to date(Dec. 2007).

Reference 16S rRNA gene sequences of vaginal origin were aligned and arranged in BioEdit [13] with Clustal W and with the Mega3 [14] software. The signatures of the V1 and V3 regions were checked for their ability to discriminate to the species level.

**Figure 2:**
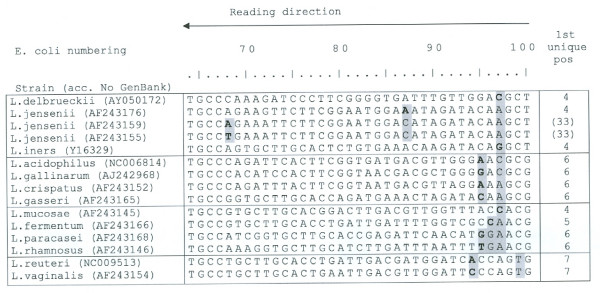
**Nucleotide sequence of the V1 region base 100–75 of the 16S rDNA in *Lactobacilli *from normal vaginal flora in women undergoing IVF treatment. **E. coli numbering for reference strains[11].

**Figure 3:**
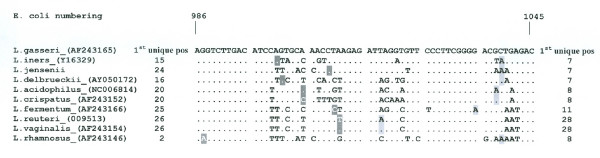
**Nucleotide sequences of the entire V3 region of 16S rDNA for the nine *Lactobacillus *strains obtained from normal vaginal flora in women undergoing IVF treatment. **E. coli numbering for reference strains[11].

The minimal length of discrimination between sequences of reference strains is for the V1 region described in Fig. 2. Positions refers to *E. coli *numbering[11]. The sequences of the V3 region, as shown in Fig. 3, are sufficiently varied to distinguish among all identified species in the study in both sense and antisense directions, however some of the other strains presented in Fig. 2 demonstrate identical sequences in the V3 region. This is e.g. the case comparing the signatures of *L. gasseri and L. johnsonii*, which indicates that the signature of V1 is necessary to group the strains.

For the V1 region, the signature of the first seven nts proved sufficient to discriminate all strains in the study as well as the sequences of reference strains of vaginal origin. There was one exception, namely *L. casei and L. (casei subspecies) paracasei *that also share identical sequences for both V1 and V3 region.

For the sense direction of the V3 region, a signature length of 26 nts was required to group the isolates. For the antisense direction of the V3 region, 11 nts was sufficient, except for separating *L. rhamnosus *and *L. vaginalis *where 28 nts were needed.

## Results and Discussion

In order to find signatures for the lactobacilli included in the study, 184 out of 186 lactobacillus isolates were subjected to PCR and pyrosequencing of the 16S rRNA gene regions V1 and V3.

Three sequences were obtained for each strain subjected to PCR and pyrosequencing, one for the V1 region and two complementary sequences for the V3 region. All were categorized separately and matched with the sequence of the reference strain of each species (for *L. jensenii *also two subtypes). Of a possible 552 sequences, 519 were suitable for sequence analysis.

Tentative assignment of all vaginal strains to individual "species-like" categories was now possible by estimating the required length of signatures.

I. For the V1 region, only the first eleven nts were required to make a unique match of each group with one of the chosen published sequences of vaginal strains.

II. Whenever more than 30 matching nts in each direction of the two V3-sequences were obtained, that sequence was assumed to be the true sequence of the complete V3 region as well as if there was a complete match of all the 60 nts of the region. This was the case for most strains.

III. Similarity was also assumed when there was a match of at least 30 nts in either sense or antisense direction of the V3 region.

A match fulfilling the criteria of highest similarity (I and II) was obtained in 109 isolates.

When I or II was fulfilled, it was assumed to be the second best identity and this was the case in 41 isolates.

Third grade of identification (I or III) was found in 25 isolates.

The 8 remaining isolates did not yield unique identity *per se*. These could be categorized by other means. Three of these strains originated from patient 17 who demonstrated a predominant flora of lactobacilli growing only on horse blood agar. Though not fulfilling any of the criteria stated above, for the purpose of the study at hand, these strains are assumed to be *L. iners*-like.

Three strains originated from one sample derived from patient 7. All three colonies yielded sequences matching *L. gasseri *or *L. vaginalis*, but varying within one and the same template from a specific colony. This sample was assumed to be impure and to consist of a mix of two separate strains. Still two samples from two different patients demonstrated the same type of discrepancy. All these strains were assigned to the most probable species when we defined the results from other colonies originating from the same patient.

The signature matching *L. jensenii*-like strains could be further subtyped into three different signature groups, each matching a strain previously published in PubMed [8]. As regards the ATCC strains listed, they differed in the V1 region only, with a variation of nts in the same two positions of the subtypes; one of which had a variation in a third position (Fig 2). Each patient exhibited only one of the subtypes in all obtained signatures.

For signature matching of *L. gasseri*, a homology was found in the V1 region sequence of one sample from patient 11; this was from position 87 with an exchange of A for G. For the V3 region there was an exchange of T for A in pos 1023 in one sequence of the same sample, as well as in one sequence of patient 4 and in all the sequences of patient 7.

The V1 and V3 regions of the16S rDNA in the 184 strains of *Lactobacilli *included in our study, thus showed extremely conserved sequences when compared with published sequences of *Lactobacilli *from the same niche, i.e. the human vagina. The signatures of the V1 and V3 regions also appeared remarkably consistent in each group. In addition to the previously published homologies in *L. jensenii *[8], we found, in some strains, base exchanges in the signatures that matched *L. gasseri*. No other obtained signature differed from the signature of the chosen reference strain of each group, not even when 50 nts or more were read, which was the case for most signatures. This indicates that pyrosequencing is a highly accurate and reproducible method.

A recent DNA probe study from the USA [15], presents two separate types of *L. crispatus *which we were not able to resolve in our study.

In 8 isolates of 184 we could not fulfill the stated criteria to group the isolates to one of the eleven reference-strain groups. Five gave contradictory results within the same sample, i. e. the signature of the V1 region and V3 region respectively were grouped differently. The most probable reason is that these samples contained a mix of two different species.

The signatures of three other isolates were too short to fulfill the criteria for matching.

These three derived from patient 17, and the remaining 12 isolates, even those selected from cultures of the same sample, were nonetheless designated as *L. iners*.

Nine reference sequences in Table 1 represent all *Lactobacilli *whole genome sequences completed as of December 2007. In these strains, the number of 16S rRNA genes varies from four in *L. acidophilus *to nine in *L. delbrüeckii (subsp. delbr.)*. All genes are identical in the V1 and V3 regions (for all reference strains representing these species) to the genes of the strains actually found in the study, except *Lactobacillus reuteri *where the sequences vary in three of the genes in the V1 region and in one in the V3 region.

**Figure 4:**
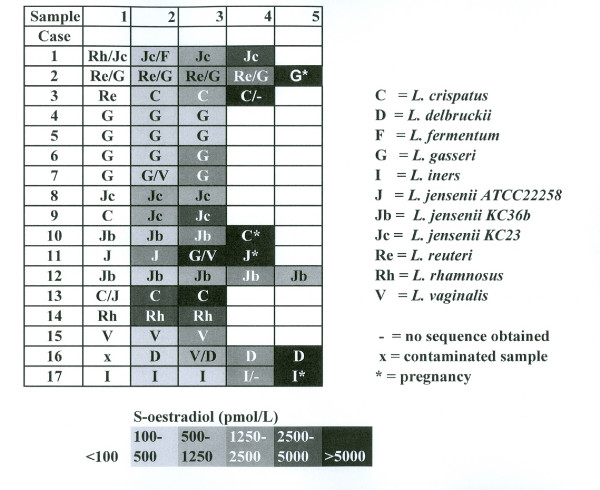
Vaginal *Lactobacilli *and estrogen levels found in women undergoing IVF treatment.

Based on our signature matching of the dominant *Lactobacilli*, the ecological niche of human vagina was monitored in a population of female partners of infertile couples, but otherwise healthy women of childbearing age. The signature-matched categorization of vaginal isolates into species-like categories led to inclusion of 182 of the 184 isolates in the overall study data (Fig. 4). When all three strains from a cultured sample were identical, only the species is indicated in each box. When two strains matched one species, and the third strain differed, the two-strain identification is denoted first (i. e. Re/G means that two of the signatures matched the sequence of *L. reuteri *and the third signature matched the sequence of *L. gasseri*). In two strains, the signatures of the V1 region and the V3 region did not match the sequences of any single reference strain.

Earlier studies of the vaginal flora have demonstrated other species, but in some the strains were derived from culture collections where the origin either was not stated or was not properly defined [1]. However for the past decade, most studies on normal vaginal flora with PCR-based methods have demonstrated the presence and dominance of *L. crispatus, L. gasseri and/or L. jensenii*. These results are consistent with the result of the study at hand as well as our earlier studies of the dominant flora of women with normal vaginal flora. Some studies have not taken into account whether the bacteria found was representative of the dominating flora or not, and some reports have simply stated that the flora is considered to be normal with no further specification[1]. Moreover, several recent studies that examined the vaginal flora with DNA-probes present extensive lists of bacterial DNA clones, of which some are not possible to cultivate [16-22]. The role of *Atopobium vaginae*, *Megasphera *and other species documented in these studies, and their relation to normal flora remains unclear.

In the study at hand, 10 of 17 patients continued to exhibit *L. crispatus, L. gasseri and/or L. jensenii *with little variation, the species dominating the flora of healthy women as shown in our previous studies (Vasquez 2002, Tärnberg 2002), cases 4–13,17. The flora of three patients was however dominated by *L. delbrüeckii, L. rhamnosus or L. vaginalis*. One patient had a dominance of *L. iners*. The remaining three had an initial flora dominated by *L. rhamnosus *or *L. reuteri*. With rising estrogen levels, the make-up of the flora altered to become dominated by one of the three species of normal vaginal flora. Thus three cases (14–16) are dominated by species that are not normally found dominating the flora of healthy women. All the samples of these women were collected within a time span of less than 20 days. This might be too short a time for a shift to the species normally found. The changes occuring in the flora of the three first patients might merely be an indicator that the flora with rising estrogen levels shifts, from being dominated with species not normally found in healthy floras, to a flora normally found in healthy women. This issue has to our knowledge never been addressed before.

*L. iners *was frequently found in a recent study of vaginal flora [23] and in three studies [17,21,24]*L. iners *was found in vaginas with abnormal flora. This raises the question whether *L. iners *should indeed be regarded as a member of the normal flora in vaginal fluid, considered to date to be dominated by *L. crispatus, L. gasseri *and *L. jensenii *[1]. Of special interest therefore, are three of the patients who were excluded from our study. Two of the patients originally recruited were found to have a BV infection and were treated with metronidazole 2 g in single dose. One week later, each exhibited normal vaginal flora, according to Amsel's and Nugent's criteria, and IVF treatment could start. One of them returned to the clinic once and the Gram-stainable flora had not changed. The third presented initially an altered flora (dominated by Gram positive cocci) that converted with rising estrogen levels to a flora dominated by Gram-positive rods, i. e. normal vaginal flora. Since the three randomly selected colonies from the flora of these patients only grew on horse blood agar, we progressed with culture and pyrosequencing of one sample from each patient. They all matched the sequences of *L. iners *[25]. This is in accordance with a recent study from Ferris et al [16] and supports the findings that *L. iners *might be the first *Lactobacillus *species to establish after BV or is associated with BV as such. In studies including patients infected with BV, the most prevalent *Lactobacillus *species is *L. iners *[16,21,24]. *L. iners *might have a position as the species establishing primarily after BV, occupying the space of the mucous membrane lining the vagina, producing lactate and thus restoring acid pH to create a milieu favorable for the overgrowth of the normally dominant *Lactobacillus *species. Some support for this idea can be found in the comparison of samples of fifteen patients participating in our first two studies [6,7], (separated by a three-year interval) that show consistently dominating species in eleven women. The vaginal flora of two women changed from dominance by *L. gasseri *to *L. crispatus *and *L. jensenii *respectively. One instance of changed dominating flora was from L. crispatus to *L. gasseri *and one from *L. iners *to *L. crispatus *(unpublished data). Two recent studies of vaginal flora relate such change to corresponding hormonal changes. In the first study, the normal flora of women in early pregnancy is described, but lactobacilli not cultivable on MRS agar are excluded [26]. The other study does not distinguish among different species of lactobacilli [27].

In conclusion, signature matching of nts in the V1 and V3 regions of 16sRNA genes is a discriminative tool for the study of vaginal *Lactobacilli *with a possibility to reliably subtype certain species. The method can be used to follow the Lactobacillus flora under differing physiological conditions.

## Authors' contributions

TJ carried out the molecular genetic studies, participated in the sequence alignment and drafted the manuscript. UF participated in the design of the study and participated in the sequence alignment. TJ and UF conceived of the study, and participated in its design and coordination. TJ and UF read and approved the final manuscript.
